# Synthesis, Characterization, and Antibacterial Studies of Mixed Ligand Dioxouranium Complexes with 8-Hydroxyquinoline and Some Amino Acids

**DOI:** 10.5402/2011/168539

**Published:** 2011-09-27

**Authors:** Sunil S. Patil, Ganesh A. Thakur, Manzoor M. Shaikh

**Affiliations:** ^1^Department of Chemistry, Changu Kana Thakur Arts, Commerce and Science College, New Panvel, Raigad, Maharashtra 410206, India; ^2^Department of Chemistry, Mahatma Phule Arts, Science and Commerce College, Panvel, Raigad, Maharashtra 410206, India

## Abstract

Mixed ligand complexes of dioxouranium (VI) of the type [UO_2_(Q)(L)*·*2H_2_O] have been synthesized using 8-hydroxyquinoline (HQ) as a primary ligand and amino acids (HL) such as L-threonine, L-tryptophan, and L-isoleucine as secondary ligands. The metal complexes have been characterized by elemental analysis, electrical conductance, magnetic susceptibility measurements, and spectral and thermal studies. The electrical conductance studies of the complexes indicate their nonelectrolytic nature. Magnetic susceptibility measurements revealed diamagnetic nature of the complexes. Electronic absorption spectra of the complexes show intraligand and charge transfer transitions, respectively. Bonding of the metal ion through N- and O-donor atoms of the ligands is revealed by IR studies, and the chemical environment of the protons is confirmed by NMR studies. The thermal analysis data of the complexes indicate the presence of coordinated water molecules. The agar cup and tube dilution methods have been used to study the antibacterial activity of the complexes against the pathogenic bacteria *S. aureus, C. diphtheriae, S. typhi,* and *E. coli*.

## 1. Introduction

It is well known that mixed ligand ternary complexes of some metals play an important role in the activation of enzymes [1]. It is studied that mixed ligand complexes are biologically active against pathogenic microorganisms [2, 3]; further, metal complexes, which include 8-hydroxyquinoline as a primary ligand, exhibit biological activity [4]. Ternary complexes containing an amino acid as a secondary ligand have a significance as they are potential models for enzyme metal ion substrate complexes [5]. Numerous uranium complexes and their mixed chelates have been studied [6, 7]. A large number of complexes with varying geometries of dioxouranium(VI), UO_2_^2+^ oxocations are possible [8]. The coordination numbers ranging from 7 to 12 for metal chelates of UO_2_(VI) and Th(IV) have been reported [9, 10]. Recently, it was stated that the UO_2_(VI) complexes show antimicrobial activity [11, 12].

This paper reports the synthesis, characterization, and antibacterial studies of mixed ligand dioxouranium(VI) complexes prepared with 8-hydroxyquinoline as a primary ligand and amino acids such as L-threonine, L-tryptophan, and L-isoleucine as secondary ligands. These complexes have been screened for their antibacterial properties against the pathogenic bacteria *S. aureus*, *C. diphtheriae*, *S. typhi, *and *E. coli.*

## 2. Materials and Methods

### 2.1. Materials

Analytical grade uranyl nitrate hexahydrate was used as such without further purification. L-threonine, L-tryptophan, L-isoleucine, and 8-hydroxyquinoline were obtained from S.D. Fine Chemicals, Mumbai. Solvents like ethanol and dimethylformamide and laboratory grade chemicals whenever used were distilled and purified according to standard procedures [13, 14].

### 2.2. Preparation of Mixed Ligand Complexes

Mixed ligand dioxouranium(VI) complexes were prepared from uranyl nitrate hexahydrate, 8-hydroxyquinoline (HQ) as a primary ligand, and different amino acids such as L-threonine, L-tryptophan, and L-isoleucine as secondary ligands.

To an aqueous solution (10 mL) of uranyl nitrate hexahydrate (502 mg, 1 mmol), ethanolic solution (10 mL) of 8-hydroxyquinoline (145 mg, 1 mmol) was added. The mixture was stirred and kept in a boiling water bath for 10 min. To this hot solution, an aqueous solution (10 mL) of amino acid (1 mmol) was added with constant stirring. The mixture (1 : 1 : 1 molar proportion) was again heated in a water bath for 10 min till the temperature reached to 50°C. The complexes were precipitated by raising the pH of the reaction mixture by adding diluted ammonia solution. The mixture was cooled, and solid complex obtained was filtered and washed with water followed by ethanol. The complexes thus prepared were dried under vacuum and were used for further studies.

### 2.3. Instrumentation

The complexes were analyzed for C, H, and N contents on Thermo Finnigan Elemental Analyzer Model no. FLASH EA 1112 Series at the Department of Chemistry, I.I.T., Mumbai. Metal content was estimated gravimetrically by standard procedure [15]. The molar conductance values were measured in DMF (10^−3^M) on an Equiptronics Autoranging Conductivity Meter Model No. EQ-667. Room temperature magnetic susceptibilities were measured by a Guoy method using Hg[Co(SCN)_4_] as a calibrant at the Department of Chemistry, I.I.T., Mumbai. The electronic absorption spectra of all the complexes in DMF solution (10^−4^M) in the ultraviolet and visible region were recorded on Shimadzu UV/VIS-160 Spectrophotometer. FT-IR spectra were recorded in KBr disc on a Perkin-Elmer FT-IR spectrophotometer Model 1600 at Department of Chemistry, I.I.T., Mumbai. NMR spectra were recorded on JEOL-300 MHz instrument using TMS as an internal standard at The Institute of Science, Mumbai. Thermal Analysis (TG and DTA) were carried out in controlled nitrogen atmosphere on a Perkin-Elmer Diamond TG-DTA Instrument at Department of Chemistry, I.I.T., Mumbai by recording the change in weight of the complexes on increasing temperature up to 900°C at the heating rate of 10°C per minute.

### 2.4. Antibacterial Screening

#### 2.4.1. Agar Cup Method

In the agar cup method, a single compound can be tested against a number of organisms or a given organism against different concentrations of the same compound. The method was found suitable for semisolid or liquid samples and was used in the present work. In the agar cup method, a plate of sterile nutrient agar with the desired test strain was poured to a height of about 5 mm and allowed to solidify, and a single cup of about 8 mm diameter was cut from the center of the plate with a sterile cork borer. Thereafter, the cup was filled with the sample solution (1000 *μ*g/mL) in dimethylsulphoxide, and the plate was incubated at 37°C for 24 h. The extent of inhibition of growth from the edge of the cup was considered as a measure of the activity of the given compound. By using several plates simultaneously, the activities of several samples were quantitatively studied.

#### 2.4.2. Tube Dilution Method

The test compound (10 mg) was dissolved in dimethylsulphoxide (10 mL) so as to prepare a stock solution of concentration 1000 *μ*g/mL. From this stock solution, aliquots of the ranges 5, 10, 15,…, 250 *μ*g/mL were obtained in test broth.

The test compounds were subjected to *in vitro *screening against *Staphylococcus aureus*, *Corynebacterium diphtheriae*, *Salmonella typhi, *and* Escherichia coli* using Muller Hinton broth as the culture medium.

Bacterial inoculums were prepared in sterilized Mueller Hinton broth and incubated for 4 h at 37°C. This was dispersed (5 mL) in each borosilicate test tube (150 × 20 mm). The test sample solution was added in order to attain a final concentration as 5 to 250 *μ*g/mL. The bacterial inoculums 0.1 cm^3^ of the desired bacterial strain (*S. aureus*, *C. diphtheriae*, *S. typhi, *and* E. coli*) containing 10^6^ bacteria/mL was inoculated in the tube. The tubes were incubated at 37°C for 24 h and then examined for the presence or absence of the growth of the test organisms. 

The lowest concentration which showed no visible growth was noted as minimum inhibitory concentration (MIC).

## 3. Results and Discussion

### 3.1. Characterization of Metal Complexes

The synthesis of mixed ligand uranyl complexes may be represented as follows:

(1) $$\begin{array}{cc} & {\text{UO}}_{2}{\left({\text{NO}}_{3}\right)}_{2}\cdot 6{\text{H}}_{2}\text{O}\;\;+\;\text{HQ}\;+\;\text{HL}\;\;\\ & \;\;\to \left[{\text{UO}}_{2}\left(\text{Q}\right)\left(\text{L}\right)\cdot 2{\text{H}}_{2}\text{O}\right]\;+\;2{\text{HNO}}_{3}\;+\;4{\text{H}}_{2}\text{O}, \end{array}$$

(where HQ is 8-hydroxyquinoline and HL is an amino acid).

All the complexes are coloured, nonhygroscopic, thermally stable solids (Table 1), indicating a strong metal-ligand bond. The complexes are insoluble in common organic solvents such as ethyl alcohol, acetone, and chloroform but are partially soluble in DMF and DMSO. 

The elemental analysis data (Table 2) of uranyl complexes are consistent with their general formulation as 1 : 1 : 1, mixed ligand of the type [UO_2_(Q)(L)·2H_2_O]. The molar conductance values (Table 2) of the complexes in DMF at 10^−3^ M concentration are found to be 0.001–0.002 Mhos cm^2^ mol^−1^ indicating their nonelectrolytic nature [16].

### 3.2. Magnetic Studies

The magnetic moment (Table 3) of the complexes was calculated from the measured magnetic susceptibilities after employing diamagnetic corrections and revealed their diamagnetic nature [17].

### 3.3. Electronic Absorption Spectra

The electronic spectra of the metal complexes in DMF were recorded in the UV-visible region. The spectra show three transitions in the range 36364–36765 cm^−1^, 29762–30303 cm^−1^, and 25253–26316 cm^−1^ ascribed to *π* → π*, n →π*, and the charge transfer transitions from the ligands to the metal, respectively.

### 3.4. Infrared Spectra

The FT-IR spectra of the metal complexes were recorded for KBr discs over the range 4000–400 cm^−1^. On the basis of the reported infrared spectra of amino acids, 8-hydroxyquinoline, and its metal complexes [18–20], some of the important bands have been assigned.

A broad band was observed in the region between 3460 and 3431 cm^−1^ due to asymmetric and symmetric O–H stretching modes and a band in the range 1600–1585 cm^−1^ due to H–O–H bending vibrations indicating the presence of coordinated water molecules further confirmed by thermal studies.

The *ν*(CO) band is observed at ~1120 cm^−1^. The position of this band undergoes variation depending on metal complex under study [21]. A strong *ν*(CO) band observed in the range between 1106 and 1105 cm^−1^ indicates the presence of oxine moiety in the complexes coordinated through its nitrogen and oxygen atoms as uninegative bidentate ligand [22]. The *ν*(C=N) mode observed at 1580 cm^−1^ in the spectra of free HQ ligand is found to be shifted to lower wave number, in the range of 1498–1497 cm^−1^ in the spectra of complexes. A negative shift in this vibrational mode on complexation indicates the coordination through ternary nitrogen donor of HQ. The in-plane and out-of-plane ring deformation modes observed at 506 cm^−1^ and 786 cm^−1^ respectively, confirm coordination through nitrogen atom of HQ with the metal.

Broad bands at 3040 and 2960 cm^−1^ due to N–H (asymmetric) and N–H (symmetric) vibrations of free amino acid moiety are shifted to higher wave numbers, in the range 3177–3140 cm^−1^ and 3050–3025 cm^−1^, respectively, in the spectra of metal complexes, suggesting coordination of the amino group through nitrogen with the metal ion.

The *ν*_asymmetric_(COO^−^) band of the free amino acid, that is, 1610–1590 cm^−1^, is shifted to lower wave number, in the range of 1571–1566 cm^−1^, and the *ν*_symmetric_(COO^−^) mode observed at ~1400 cm^−1^ in the spectra of free amino acids is found to be shifted to lower wave number 1378 cm^−1^, in the spectra of complexes indicating the coordination of the carboxylic acid group via oxygen with the metal ion [18]. 

An important feature of infrared spectra of the metal complexes with 8-HQ is the absence of band ~3440 cm^−1^ due to the O–H stretching vibration of the free O–H group of HQ [20]. This observation leads to the conclusion that complex formation takes place by deprotonation of the hydroxyl group of HQ moiety. 

The FT-IR spectra of the uranyl complexes show no absorption bands near 1352 cm^−1^ where ionic nitrate is known to absorb [23], indicating absence of ionic nitrate. Other bands observed at ~1468, ~1278, ~1035, and ~734 cm^−1^ corresponding to *ν*_1_, *ν*_4_, *ν*_2_, and *ν*_3_ vibrations agree with frequencies reported for bidentate nitrate group [24, 25]. 

The bands at 898–889 cm^−1^ and 821–820 cm^−1^ were assigned to *ν*_asymmetric_(O–U–O) and *ν*_symmetric_(O–U–O) vibrational modes of linear O=U=O moiety [26, 27]. 

Some new bands of weak intensity observed in the regions around 604 cm^−1^ and 486 cm^−1^ may be ascribed to the M–O and M–N vibrations, respectively [28]. It may be noted that these vibrational bands are absent in the infrared spectra of HQ as well as amino acids.

### 3.5. NMR Spectra

^1^H NMR spectra of complexes in DMSO exhibits a singlet at *δ* 2.8 ppm (2H; –NH_2_) due to amino group protons and broad multiplet in the region *δ* 6.6–7.8 ppm (6H; aromatic protons) due to the aromatic ring protons. The presence of water molecules in the complexes is confirmed by the appearance of a new signal around *δ* 3.5 ppm, attributed to H_2_O protons [29]. 

In case of complex with L-threonine it shows doublet at d 1.26 ppm (*J* = 7.5 Hz) for three protons of methyl group, doublet at *δ* 2.52 ppm (*J* = 7.5 Hz) for one proton of –CH, multiplet at *δ* 2.90–2.96 ppm (–CH–CH_3_) for one proton of –CH, and singlet at *δ* 10.1 ppm for hydroxyl proton which was D_2_O exchangeable.

The complex with L-tryptophan shows doublet at *δ* 2.76 ppm (*J* = 8.0 Hz) for two protons of methylene group, triplet at *δ* 2.94 ppm (*J* = 8.0 Hz) for one proton of –CH, singlet at *δ* 5.25 ppm for one proton of –NH which was D_2_O exchangeable, and multiplet at *δ* 6.6–7.2 ppm (indole ring protons for aromatic protons.

The complex with L-isoleucine shows triplet at *δ* 0.93 ppm (*J* = 7.0 Hz) (–CH_2_–CH_3_) for three protons of methyl group, doublet at *δ* 1.15 ppm (*J* = 7.0 Hz) (–CH–CH_3_) for three protons of another methyl group, multiplet at *δ* 1.50–1.57 ppm for two protons of –CH_2_, another multiplet at *δ* 1.60–1.68 ppm for one proton of –CH, and doublet at *δ* 2.92 ppm (*J* = 7.0 Hz) for one proton of another –CH.

### 3.6. Thermal Studies

The TG and DTA studies of the uranyl complexes have been recorded in the nitrogen atmosphere at the constant heating rate of 10°C per minute. 

The TG of the uranyl complexes shows that they are thermally quite stable to varying degree. The complexes show gradual loss in weight due to decomposition by fragmentation with increasing temperature as presented in Table 4. All the complexes show similar behavior in TG and DTA studies. The thermogram of these complexes shows the loss in weight corresponding to two water molecules in the temperature range 110–190°C, followed by weight loss due to amino acid moiety in the range 250–445°C. The final step of the decomposition observed in the range 450–690°C corresponds to the weight loss of HQ moiety present in the complexes. 

The DTA of the complexes displays an endothermic peak in the range 110–190°C which indicates the presence of coordinated water molecules. As the temperature is raised, the DTA curve shows a small exotherm in the range 250–445°C and a broad exotherm in the range 450–690°C attributed to decomposition of amino acid and 8-hydroxyquinoline moieties present in the complexes, respectively. The formation of a broad exotherm is possibly due to simultaneous decomposition of ligand moieties and their subsequent oxidation to gaseous products like CO_2_ and H_2_O and so forth [17]. 

Like most of the metal organic complexes, these complexes also decompose to a fine powder of metal oxide, that is, UO_2_. The constant weight plateau in TG after 700°C indicates completion of the reaction. The UO_2_ form was confirmed by X-ray diffraction pattern of the decomposed product [17]. 

**Figure 1:**
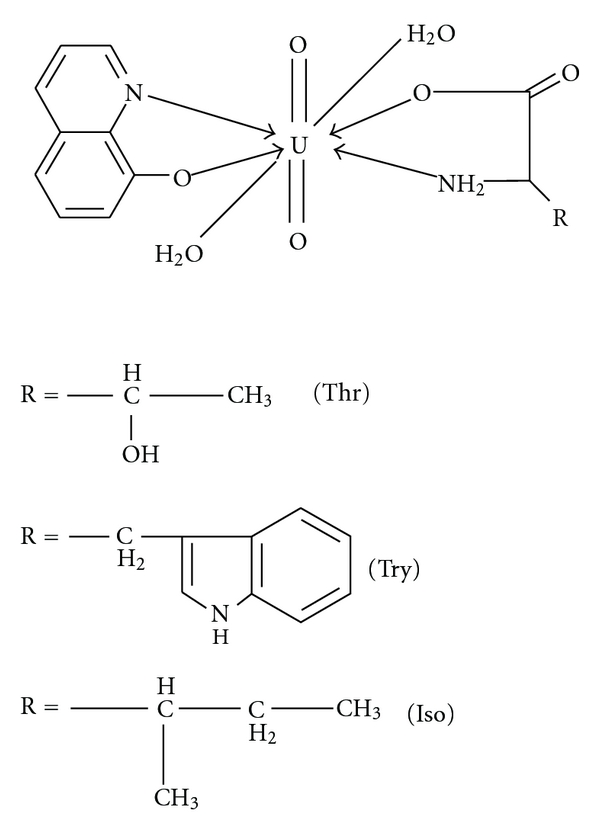
Proposed structures and bonding for the uranyl complexes.

On the basis of the physicochemical studies, the bonding and structure for the uranyl complexes may be represented as shown in Figure 1. 

### 3.7. Antibacterial Studies

All the metal complexes were screened against *Staphylococcus aureus*, *Corynebacterium diphtheriae*, *Salmonella typhi, *and* Escherichia coli*.

The studies based on agar cup method revealed that the complexes are more active against *S. typhi *and *E. coli *and less active against *S. aureus* and *C. diphtheria *(Table 5). 

The minimum inhibitory concentration (MIC) of ligand and the metal salts ranges between 50 and 110 *μ*g/mL while that of metal complexes ranges between 5 and 25 *μ*g/mL (Table 6). The complexes are found to be more active against *S. typhi *and *E. coli *as compared to *S. aureus* and *C. diphtheria*. As compared to standard antibacterial compound tetracycline, the complexes show moderate activity against selected strains of microorganisms [30]. 

The results show that, as compared to the activity of metal salts and free ligand, the metal complexes show higher activity. The activity of metal complexes is enhanced due to chelation. The chelation reduces considerably the polarity of the metal ions in the complexes, which in turn increases the hydrophobic character of the chelate and thus enables its permeation through the lipid layer of microorganisms [31].

## 4. Conclusions

Based on the above results, the following conclusions may be drown.

The higher decomposition temperatures of the complexes indicate a strong metal-ligand bond, and electrical conductance studies show nonelectrolytic nature of the complexes, respectively. Magnetic studies indicate diamagnetic nature of the complexes. Electronic absorption spectra of the complexes show intraligand and charge transfer transitions, respectively. IR spectra show bonding of the metal ion through N- and O- donor atoms of the two ligands. ^1^H NMR study reveals the chemical environment of protons and presence of water molecules in the complexes. Thermal analysis confirms the presence of coordinated water molecules. 

On the basis of the above results, coordination number eight is proposed for uranyl complexes.

The antibacterial study shows that complexes are found to be more active against* S. typhi *and *E. coli *as compared to *S. aureus* and *C. diphtheria*.

Compared to standard antibacterial compound, tetracycline, the complexes show moderate activity against the selected strains of microorganisms.

## Figures and Tables

**Table 1:** Colour, decomposition temperature, and pH of the uranyl complexes.

Sr. no.	Complex	Colour	Decomposition temperature(°C)	pH
(1)	[UO_2_(Q)(Thr)·2H_2_O]	Light Brown	265	7.00
(2)	[UO_2_(Q)(Try)·2H_2_O]	Light Brown	257	7.00
(3)	[UO_2_(Q)(Iso)·2H_2_O]	Light Brown	250	7.00

Where Q represents the deprotonated primary ligand 8-hydroxyquinoline, Thr, Try, and Iso represent deprotonated secondary ligands L-threonine, L-tryptophan, and L-isoleucine, respectively.

**Table 2:** Empirical Formula, Molecular Weight, Elemental Analysis Data and Molar Conductance of Uranyl Complexes.

Sr. no.	Complex	Empirical formula	Molecular weight	Elemental analysis Found (Calculated)				Molar conductance Mhos·cm^2^ moL^−1^
				%M	%C	%H	%N	
(1)	[UO_2_(Q)(Thr)·2H_2_O]	UC_13_H_18_N_2_O_8_	568.31	41.81 (41.88)	27.40 (27.45)	3.15 (3.17)	4.91 (4.93)	0.001
(2)	[UO_2_(Q)(Try)·2H_2_O]	UC_20_H_21_N_3_O_7_	653.42	36.43 (36.43)	36.72 (36.73)	3.20 (3.21)	6.41 (6.43)	0.001
(3)	[UO_2_(Q)(Iso)·2H_2_O]	UC_15_H_22_N_2_O_7_	580.37	41.00 (41.01)	31.01 (31.01)	3.76 (3.79)	4.81 (4.82)	0.002

**Table 3:** Magnetic susceptibility data of uranyl complexes (−10^−6^ c.g.s. units).

Sr. no.	Complex	*X*_*g*_	*X*_*m*_	*μ*_eff_ B.M.
(1)	[UO_2_(Q)(Thr)·2H_2_O]	0.6844	389.00	Diamagnetic
(2)	[UO_2_(Q)(Try)·2H_2_O]	0.9499	620.69	Diamagnetic
(3)	[UO_2_(Q)(Iso)·2H_2_O]	0.9855	571.99	Diamagnetic

**Table 4:** Thermal data of uranyl complexes.

Sr. no.	Complex	Decomposition temperature (°C)	Temperature range (°C)	% Weight loss		Decomposition product
				Found	Calculated	
(1)	[UO_2_(Q)(Thr)·2H_2_O]	250	110–190	06.00	06.04	[UO_2_(Q)(Lys)]
			250–360	24.20	24.35	[UO_2_(Q)]
			425–550	24.10	24.19	[UO_2_]
(2)	[UO_2_(Q)(Try)·2H_2_O]	260	110–190	06.00	06.18	[UO_2_(Q)(Asp)]
			260–360	22.52	22.67	[UO_2_(Q)]
			430–520	24.51	24.73	[UO_2_]
(3)	[UO_2_(Q)(Iso)·2H_2_O]	230	110–160	06.31	06.31	[UO_2_(Q)(Cys)]
			230–380	21.02	21.04	[UO_2_(Q)]
			460–585	25.00	25.25	[UO_2_]

**Table 5:** Antibacterial activity (mm) of uranyl complexes by agar cup method.

Sr. no.	Complex	Test			
		*S. aureus*	*C. diphtheriae*	*S. typhi*	*E. coli*
(1)	[UO_2_(Q)(Thr)·2H_2_O]	12	12	20	26
(2)	[UO_2_(Q)(Try)·2H_2_O]	12	14	23	18
(3)	[UO_2_(Q)(Iso)·2H_2_O]	13	12	26	25
(4)	Tetracycline	30	25	26	26

**Table 6:** MIC data of uranyl complexes.

Sr. no.	Complex	MIC (*μ*g/mL)			
		*S. aureus*	*C. diphtheriae*	*S. typhi*	*E. coli*
(1)	[UO_2_(Q)(Thr)·2H_2_O]	20	20	5	10
(2)	[UO_2_(Q)(Try)·2H_2_O]	20	25	10	10
(3)	[UO_2_(Q)(Iso)·2H_2_O]	20	20	10	5
(4)	UO_2_(NO_3_)_2_·6H_2_O	50	50	100	100
(5)	8-hydroxyquinoline	50	50	110	100
(6)	Tetracycline	1.5	2.0	1.5	2.5
